# Long‐Term Treatment Outcome and Recidivism Patterns Among Intimate Partner Violence Perpetrator Typology: A 15‐Year Follow‐Up Study

**DOI:** 10.1002/cpp.70142

**Published:** 2025-08-17

**Authors:** Natalia Redondo, Román Ronzón‐Tirado, Marina J. Muñoz‐Rivas, Jose Luis Graña

**Affiliations:** ^1^ Faculty of Psychology, Department of Biological and Health Psychology Universidad Autónoma de Madrid Madrid Spain; ^2^ Faculty of Health Sciences, Department of Psychology Universidad de Deusto Bilbao Spain; ^3^ Faculty of Psychology, Department of Clinical Psychology Universidad Complutense de Madrid Madrid Spain

**Keywords:** latent class analysis, long‐term recidivism, perpetrators of intimate partner violence, survival analysis, typology

## Abstract

Current studies on intimate partner violence (IPV) conclude that IPV perpetrators are a heterogeneous group, with substantially different profiles and different relevant clinical variables, with a differential response to the psychological treatment programmes that they take part in, measured through criminal recidivism. However, most studies look only at these offenders' short‐term recidivism. The aim of this longitudinal study was, on the one hand, to replicate a typology based on the risk of recidivism in a sample of 484 court‐referred partner‐violent men and, on the other hand, to analyse long‐term criminal recidivism in these aggressors, depending on the typology found. For this purpose, a 15‐year longitudinal follow‐up was conducted on 484 court‐referred partner‐violent men after they had participated in a cognitive‐behavioural psychological treatment programme. The results corroborated the existence of three subtypes of aggressors: those with high risk, medium risk and low risk, as well as different patterns of recidivism depending on the profile identified at the beginning of the treatment. It was concluded that recidivism was higher in the first year of follow‐up, as well as the fact that the aggressors at greatest risk were the subgroup with the highest level of long‐term recidivism. These results highlight the heterogeneity existing in this type of aggressor, as well as the need to adapt psychological treatment programmes in line with the initial characteristics of the participants.

## Summary

- Effective psychological treatment for intimate partner violence perpetrators requires adapting programmes to the varying needs and profiles of participants.
- The period of highest risk for recidivism occurs in the year following completion of therapy. It would be advisable to conduct follow‐up, both therapeutic and legal/police‐based, especially during that year.
- Partner violence perpetrators with higher psychopathological deviation and associated problems are the group most likely to reoffend. It would be beneficial to extend the duration and content of treatment programmes for this profile of aggressors and establish more exhaustive and prolonged follow‐up, both therapeutically and through the justice system.

According to the World Health Organization (2021), prevalence rates of women victims of Intimate Partner Violence (IPV) are still very high and of considerable concern. All this, together with the serious consequences of IPV at the physical, psychological, judicial and family levels, means that this problem continues to be considered a social and public health problem of the first magnitude. However, despite being the focus of extensive scientific research, many unresolved issues remain regarding this problem (Babcock et al. 2024). One of the most frequently studied aspects is the effectiveness of treatment programmes for IPV perpetrators. These programmes have been developed with the aim of rehabilitating offenders and reducing both IPV levels and recidivism rates. Nevertheless, the available data do not support the conclusion that these programmes are having the significant impact one might expect, likely due to certain limitations that still need to be addressed. One such limitation is the high recidivism rates observed among participants. Recidivism, as a long‐term indicator of programme effectiveness, is particularly difficult to assess, as monitoring violent behaviours over extended periods poses considerable challenges. In their classic Babcock et al. 2004 study, Babcock et al. conducted a meta‐analysis of IPV perpetrator treatment programmes. The authors reviewed 22 published studies and examined the impact of programme participation on police‐reported recidivism rates. They concluded that the overall effect size (Cohen's *d*) of the various programmes analysed was small, ranging from 0.09 to 0.34. Their general conclusion was that men who underwent therapy were 5% less likely to reoffend. While this is certainly a positive finding, it falls short of expectations given the substantial resources and efforts invested in these types of interventions. In another meta‐analysis focused on the recidivism of IPV perpetrators, Olver et al. (2011) found that individuals who failed to complete treatment had a higher likelihood of reoffending. However, the moderating variables underlying the relationship between treatment dropout and recidivism remain unclear. It appears that initial individual characteristics—such as being younger, unemployed, having a low level of education, a history of prior partner violence, antisocial personality traits or substance abuse—are associated with higher dropout rates, and consequently, with higher recidivism. After years of research, significant progress has been made as regards the consensus over the bases and theoretical foundations of the key factors that influence the effectiveness of intervention programmes, pointing out that the results obtained may be significantly affected by the tendency to work with aggressors on a homogeneous basis. From there, studies on IPV perpetrator typologies emerge, which have seen great development in recent years (Alexander and Johnson 2023; Peters et al. 2023; Teva et al. 2022). Oğuztüzün et al. (2023) developed a meta‐analysis where they analysed the relationship between the levels and type of pretreatment violence by the participants and the effectiveness of these interventions, finding that the higher the levels of physical violence, the more effective the treatment programmes with IPV perpetrators, compared with other types of violence. In this context, the interest arises in analysing the effectiveness of intervention programmes in line with the typologies of the aggressors, in order to discriminate between their differential effect on the long‐term recidivism of the participants, depending on the characteristics of the level of aggression perpetrated, their personality and/or substance use.

One of the most important typologies and one that has gathered the greatest empirical support to date is the one proposed by Holtzworth‐Munroe and Stuart (1994), who postulated the existence of three subtypes of partner‐violent men from lower to higher levels of IPV. From this first typology, others have been developed, although all of these concur as to the inclusion of the severity and frequency of the violence exercised, the presence of psychopathology and the criminal history of the aggressor (Cavanaugh and Gelles 2005; Peters et al. 2023), with the classification into three subtypes of IPV perpetrators being the most frequently found (Alexander and Johnson 2023). Specifically in Spain, Graña et al. (2014), in a sample of partner‐violent offenders in court‐ordered treatment or psychological treatment, found three different subtypes depending on IPV levels and the presence of psychopathology: (1) the low‐level violence and psychopathology group, which showed lower levels of psychopathology and lower frequency of IPV; (2) the moderate‐level violence and psychopathology group, which was in between the two groups; and (3) the high‐level violence and psychopathology group, which showed a higher level of deviation in the psychopathological characteristics analysed and greater severity and frequency of IPV. In addition, analysing the response to treatment of the three subtypes, they found significantly lower rates of recidivism in police officers (1 year after the end of therapy) in the three subtypes in treatment, compared with the control group (Graña et al. 2017). Similarly, the low‐risk group was the one with the lowest recidivism rate, although no significant differences were found between the three subtypes in terms of recidivism, which, in any case, was measured only in the short term. Along these lines, different studies have already concluded that the response to treatment of this type of aggressor varies, depending on the previous characteristics they presented at the beginning of therapy, taking into account different profiles and relevant clinical variables (Cunha et al. 2022; Redondo, Muñoz‐Rivas, et al. 2023; Vargas et al. 2020; Verdugo et al. 2025).

In the light of the existing data in the scientific literature, and in order to clarify to what extent different subtypes of partner‐violent men respond differently to the psychological intervention over the years, the objectives of this study were as follows: (a) to identify a typology in a sample of 484 court‐referred partner‐violent men under psychological treatment, classifying participants into different subgroups, with the aim of replicating the typology found by Graña et al. (2014); (b) to analyse the efficacy of the treatment programme depending on the type identified, with the aim of determining the effect of the participants' previous characteristics in their response to treatment and its effect over time. Criminal justice recidivism was an outcome of the effectiveness of the programme, which was measured taking the number of IPV‐related arrests after completion of the treatment programme, in accordance with comprehensive follow‐up data from the Spanish Interior Ministry's IPV monitoring system (Sistema VioGén; López‐Ossorio et al. 2016). The system contains data on all cases of gender violence nationwide and logs any new complaints that may be filed. As well as recording each case, the database contains information on noncompliance with court orders (e.g., restraining orders) reported by any of the institutions or professionals involved in protecting victims.

The starting hypotheses were as follows: (a) There are three subtypes of IPV perpetrators based on their initial psychological characteristics, thus replicating the typology proposed by Graña et al. (2014); (b) the different subtypes of IPV perpetrators respond to treatment in a differentiated manner, with those who present a higher level of violence towards the partner and greater psychopathological deviation at the beginning of the programme being the most likely to reoffend (Cunha et al. 2022; Redondo, Muñoz‐Rivas, et al. 2023; Verdugo et al. 2025), given their high levels of violent behaviour and other relevant clinical characteristics.

## 1 Method

### 1.1 Participants

The participants in this study were court‐referred offenders from the Region of Madrid. They all had received a suspended prison sentence of less than 2 years for IPV perpetration, conditional upon completion of a mandatory psychological treatment programme under Section IV of the Spanish Gender Violence Act (Organic Law 2004), article 35. This law regulates alternative sentencing as follows: ‘Where an offender is convicted of a gender violence offence, (…) the Judge or Court shall order the offender to attend a specific re‐education and/or psychological treatment program (…)’. The inclusion criteria were as follows: understanding spoken and written Spanish, not having a serious drug and/or alcohol consumption problem that would interfere with the treatment programme and not presenting psychotic symptoms. The final sample of the study was composed of 484 men with a mean age of 38.12 years (SD = 10.16). Regarding the level of education, 41.6% (*n* = 199) had primary education, 41.4% (*n* = 198) had completed secondary education, and 16.3% (*n* = 78) had undergraduate or postgraduate education. Regarding the profession, most men were engaged in construction, hospitality or industrial activities (57.3%, *n* = 274), followed by those who were self‐employed professionals (9.4%; *n* = 45); those who worked in administration or business (7.8%, *n* = 37); management positions (2.1%, *n* = 10); and police officers (1.0; *n* = 5). A total of 17.4% (*n* = 83) were unemployed, and 3.3% (*n* = 16) were pensioners or retirees; 63.0% (*n* = 301) had Spanish nationality; 26.2% (*n* = 125) were from a Latin American country; 5.2% (*n* = 25) from some other European country; and 2.7% (*n* = 13) Moroccan. Concerning the offence, 83.1% were convicted of physical violence—the most frequent forms being frequent hitting, grabbing, hair pulling and shaking—whereas 16.9% were convicted of psychological violence—mainly threatening and insulting their partners.

### 1.2 Procedure

The assessment phase was carried out individually, with two therapists trained in the application of the assessment protocol. The entire assessment was performed before initiating the psychological treatment programme in which IPV perpetrators had to participate by court order. It was the first time they had participated in a programme of these characteristics. Between four and eight weekly 60‐min sessions were run with each participant, during which the following activities took place: (a) explanation of the conditions and goals of the research, granting informed consent; (b) collection of sociodemographic data and analysis of the offence committed; (c) counter‐balanced self‐administration of the scales described in the Instruments section. To minimise the social desirability of participants' responses, participants were informed that the therapists responsible for the group therapy would not have access to their responses. All questionnaires were self‐administered, as well as all the questions referring to the partner who had been the victim of the offence for which they had been convicted, who was not necessarily their partner at the time of the evaluation.

Participants had to complete a cognitive‐behavioural treatment (CBT) programme, with the content of the programme being run and specified, session by session, in a manual with which the therapists of the Graña et al. (2008) study had been trained. This programme consisted of 23 weekly 90‐min sessions conducted in 8‐patient closed‐group format by two master's degree‐level therapists. There were also weekly supervision sessions during the programme to review the session activities and intervention strategies.

Following their participation in the programme, participants were followed over 15 years (from 2009 to 2024) to record whether there had been any further IPV arrests over this time period. This data was obtained from the Comprehensive Follow‐up data in the Spanish Interior Ministry's IPV monitoring system (Sistema VioGén; López‐Ossorio et al. 2016).

This study was approved by the Ethics Committee of the Psychology Faculty at the Universidad Complutense de Madrid, on 30th May 2009. Written informed consent was obtained from all participants, all of them being informed of the aims of the research, as well as the procedure to be followed and the estimated duration of the treatment.

### 1.3 Instruments

*Sociodemographic Questionnaire*: This included questions on sociodemographic characteristics and personal variables of the participants: age, marital status, nationality, level of education and profession. The information regarding the crime was obtained through the analysis of court sentences. All the victims were women; however, their characteristics could not be included as the Spanish Gender Violence Act prohibits disclosure of such information.

*Revised Conflict Tactics Scale* (*CTS2*; Straus et al. 1996), Spanish adaptation of Loinaz et al. (2012): With a self‐administered questionnaire format, this scale assesses the frequency, prevalence and severity of aggression in intimate partner relationships. It consists of 78 items; 39 ask about the perpetration of aggressive acts and 39 about victimisation throughout the last year of cohabitation. This consisted of five subscales (bargaining, psychological, physical, sexual aggression and harm or injury) with *Cronbach's alpha* coefficients ranging from 0.79 to 0.95 (Straus et al. 1996). In this study, the alpha coefficient for mild psychological aggression was 0.78; 0.40 for severe psychological aggression; and 0.55 for both mild physical aggression and serious physical aggression. The omega coefficient was 0.78, 0.48, 0.61 and 0.60, respectively. Likewise, statistically significant correlations were found between these 4 CTS subscales and the psychological and physical aggression subscales of the AQ Aggression Questionnaire, specifically correlations between *r*(526) = 0.15 and 0.39; *p* < 0.001.

*The Aggression Questionnaire* (*AQ*; Buss and Perry 1992), Spanish adaptation by Redondo et al. (2017): The 29‐item questionnaire measures Physical Aggression, Verbal Aggression, Anger and Hostility. Test–retest reliability correlations ranged from 0.72 for the anger subscale to 0.80 for the Physical Aggression subscale (Buss and Perry 1992). In the Spanish adaptation, Cronbach's *α* was 0.80 for Physical Aggression and 0.68 for Verbal Aggression (Redondo et al. 2017). Cronbach's *α* in this study was 0.78 for Physical Aggression and 0.71 for Verbal Aggression. The omega coefficient was 0.81 and 0.73, respectively.

*The Alcohol Use Disorders Identification Test* (*AUDIT*; Saunders et al. 1993): This is a 10‐item measure of alcohol consumption, dependence and related consequences that is widely used for both research and clinical purposes. The test had good internal consistency and excellent sensitivity and specificity as an alcohol screen (Allen et al. 1997). Internal consistency in this study was *α* = 0.80; *ω* = 0.83.

*The Levenson Primary and Secondary Psychopathy Scale* (*LPSP*; Levenson et al. 1995, Spanish adaptation by Redondo, Ronzón‐Tirado, et al. 2023): This 26‐item self‐report scale measures the domains of manipulation, callousness and impulsivity. It has adequate psychometric properties in independent research (Lynam et al. 1999). In this study, the internal consistency was *α* = 0.71 and *ω* = 0.73 for primary psychopathy and *α* = 0.64 and *ω* = 0.66 for secondary psychopathy.

*The State–Trait Anger Expression Inventory 2 (STAXI‐2*; Spielberger 1988, Spanish adaptation by Miguel‐Tobal et al. 2001). The inventory consisted of 49 items that measure State Anger, Trait Anger and different forms of Anger Expression and Control. The results found in all scales and subscales of the STAXI‐2 indicated good internal consistency, with values ranging from 0.82 for Trait Anger to 0.69 and 0.67 for Anger Expression (Miguel‐Tobal et al. 2001). Cronbach's *α* in this study was 0.84 for state anger, 0.84 for trait anger, 0.73 for physical expression of anger, 0.69 for verbal expression of anger, 0.84 for internal control of anger and 0.86 for external control. The omega coefficients were 0.88, 0.85, 0.76, 0.75, 0.84 and 0.86, respectively.

*The Plutchik Impulsive Control Scale* (Plutchik and Van Praag 1989; Spanish adaptation by Rubio et al. 1998): This consists of four subscales: Planning Capacity; Emotional Control; Control of eating behaviour; spending money or maintaining sexual relations; and control over other behaviours. The reliability in the original study was 0.73 (Plutchik and Van Praag 1989) and 0.90 in the Spanish adaptation (Rubio et al. 1998). In the present study, the internal consistency was *α* = 0.71 and *ω* = 0.75.

*The Structured Clinical Interview for DSM‐IV Axis II Personality Disorders (SCID‐II*; First et al. 1999): This assesses the presence or absence of the symptoms included in the DSM‐IV for different personality disorders. In this study, only the items related to Antisocial and Borderline Personality Scales were given. The authors point out that the test–retest reliability was 0.84 for antisocial disorder and 0.37 for borderline disorder. In our study, the internal consistency was *α* = 0.79 and *ω* = 0.8.

*Criminal Justice Recidivism* (IPV new arrests): This was measured by the number of IPV‐related arrests after completion of the treatment programme, according to Comprehensive Follow‐up data from the Spanish Interior Ministry's IPV monitoring system (Sistema VioGén; López‐Ossorio et al. 2016). This database is used to measure gender violence recidivism in Spain (e.g., Redondo, Muñoz‐Rivas, et al. 2023). The system contains data on all cases of gender violence nationwide and logs any new complaints that may be filed. As well as recording each case, the database contains information on noncompliance with court orders (e.g., restraining orders) reported by any of the institutions or professionals involved in protecting victims.

### 1.4 Data Analysis

First, the internal consistency of the scales used in the study was analysed using Cronbach's alpha and MacDonald's omega. Subsequently, a latent profile analysis (LPA) was calculated to corroborate the existence of latent profiles of men convicted of gender‐based violence (*c*) who shared a significant and interpretable pattern of responses in the variables of interest observed (Ferguson et al. 2020). A total of 14 indicators were used, according to the typologies of previous aggressors of gender‐based violence (Graña et al. 2014). This typology included the frequency and severity of aggression against the partner, levels of general aggression, alcohol consumption, psychopathy, anger, impulsivity, antisocial and borderline personality.

To estimate the latent class profile, an initial LPA was estimated with one single class that served as a baseline to compare the fit of the subsequent models. Subsequently, the number (*k*) included in the model (Nylund‐Gibson and Choi 2018) was gradually increased one by one until a six‐class solution was reached. The retention of the best LPA model was taken based on the following: (1) the Bayesian Information Criteria (BIC), the Sample‐Adjusted BIC (SABIC) and the Akaike's Information Criteria (AIC) indices. Lower values for all three indices were indicators of best fit; (2) the Bootstrap Likelihood Ratio Test (BLRT) index; the significant difference between models for the Bootstrap Likelihood Ratio Test (BLRT) was interpreted as a more parsimonious indicator with better fit for the models (Masyn 2013); and (3) the relevance and theoretical interpretability of the solution found was based on the previous typologies suggested in the existing literature (Graña et al. 2014). Because there is currently no formula or calculator to estimate the sample size required to model an LPA, regarding the power and sample size requirements, we considered the current recommendations described in simulation studies on the minimum sample size to estimate an LPA (300 to 500 participants; Tein et al. 2013). In this case, 484 participants were selected. Cases where items were missing were estimated in the LPA process in Mplus, utilising full‐information maximum likelihood (FIML). Participants were removed from the analysis if the values for all variables in the study were missing (*n* = 6), with the final sample for this first analysis being *n* = 478.

Subsequently, a Kaplan–Meier analysis (Kaplan and Meier 1958) was performed to estimate the survival function of the participants (*n* = 478), for which the differences in the follow‐up time for each case, the number of men at risk of recidivism and the number of events for each time point were taken into account, the time being measured in years. Once the general trend of recidivism had been analysed, a Cox regression analysis (Cox 1972) was run to estimate the proportional risks related to belonging to the risk profile. All analyses were performed with R Statistics *r‐base* and the *survival package*.

## 2 Results

### 2.1 Analysis of Latent Profiles

A total of six LPAs were estimated to determine the underlying ranking, based on the observed responses of participants for the 14 indicators (listed in Table 2). AIC, BIC and SSABIC indices improved from the model *k* = 1 to the model *k* = 6; these being the most significant decreases in values observed between models one and two, as well as between models two and three. Also, when considering BLRT values, the *p* associated with models indicated greater fit and were more parsimonious for the *k* = 2 model, compared with the baseline model, as well as the rest of the models estimated with respect to the previous one (Table 1). As reported in previous literature, it is not uncommon for BLRT values not to reach nonsignificant values, due to the addition of new parameters, as the number of classes increases (Ferguson et al. 2020). Consequently, log‐likelihood values were plotted to identify the bend or ‘elbow’ where the log‐likelihood value of the estimated models began to decrease more gradually. In this case, this was from the three‐class model to the four‐class model, so it was considered that the three‐class model was the one that best represented the data. Additionally, the entropy value for the three‐class model was the highest, suggesting that this model was the best to differentiate the data into qualitatively different profiles.

**TABLE 1:** Fit indices for latent class models with one to six classes based on Graña et al. (2014) IPV perpetrator typology (*n* = 478).

*k* classes	AIC	BIC	SSABIC	Entropy	BLRT
1	38,812.16	38,928.97	38,840.10	—	—
2	37,686.06	37,865.45	37,728.97	87	1156.09***
3	37,123.55	37,365.51	37,181.42	0.91	592.51***
4	36,777.74	37,082.27	36,850.58	0.93	375.81***
5	36,523.41	36,890.52	36,611.21	0.90	274.84***
6	36,369.12	36,798.81	36,471.90	0.89	184.28***

Abbreviations: AIC = Akaike information criterion, BIC = Bayesian information criterion, BLRT = bootstrapped likelihood ratio test, SSABIC = sample size adjusted to Bayesian information criterion.

***p < 0.001.

Finally, with the intention of confirming the validity of the profiles found, the size of *n* for each group was compared in relation to the sizes of *n* found in previous studies that had used the same risk classification. This analysis corroborated the similarity of the sample size of the latent profiles proposed in previous studies with those found in the present study. Specifically, Graña et al. (2014) identified three risk profiles: high risk of recidivism (HRR; 7.1%), moderate risk of recidivism (MRR; 27.8%) and low risk of recidivism (LRR; 65%). Results consistent with the classification into three profiles suggested in this study: high risk of recidivism (5.2%; *n =* 25), moderate risk (27.2%; *n* = 130) and low risk (67.6%; *n* = 323). Therefore, the three‐class model was selected as the one that best represented the response patterns of the aggressors (Table 1).

### 2.2 Differences in LPA indicator Scores Between Groups

When analysing the differences in the scores for the indicators used to estimate latent profiles (Table 2), it was identified that the variables of severe psychological violence, borderline personality, general physical aggression and trait anger were the indicators with the greatest differences in scores between profiles, with effect sizes for the differences between groups equivalent to *d* = 0.80, 0.44, 0.35 and 0.35, respectively.

**TABLE 2:** Three‐profile LPA model results based on Graña et al. (2014) IPV perpetrator typology (*n* = 478).

Variable	LRR *n =* 323	MRR *n* = 130	HRR *n* = 25	ANOVA		Cohen's *d*		
				Omnibus *F* (df)	*η*_*p*_^2^	I vs. II	I vs. III	II vs. III
Mild Psychological IPV	9.21(12.20)_a_	26.74(23.32)_b_	53.24(30.19)_c_	107.84(2466)***	0.32	1.02	2.56	1.54
Severe Psychological IPV	0.70(1.69)_a_	2.91(3.77)_b_	30.75(9.75)_c_	956.85(2473)***	0.80	0.68	9.30	8.61
Mild Physical IPV	1.55(3.24)_a_	4.57(7.36)_b_	9.08(10.88)_c_	33.72(2471)***	0.12	0.57	1.42	0.85
Severe Physical IPV	0.42(1.63)_a_	0.89(2.07)_b_	1.61(3.46)_b_	6.20(2467)**	0.03	0.24	0.62	0.38
General Physical Aggression	14.30(4.24)_a_	22.83(8.18)_b_	21.58(6.83)_b_	125.05(2466)***	0.35	1.59	1.36	0.23
General Verbal Aggression	9.66(3.39)_a_	13.74(4.02)_b_	13.45(3.58)_b_	64.79(2470)***	0.22	1.12	1.05	0.07
Alcohol consumption	3.77(3.67)_a_	8.69(6.35)_b_	8.80(7.98)_b_	52.02(2455)***	0.19	1.02	1.04	0.02
Primary psychopathy	10.68(5.49)_a_	16.34(6.43)_b_	17.85(6.28)_b_	49.85(2435)***	0.19	0.97	1.23	0.26
Secondary psychopathy	6.35(3.53)_a_	12.31(4.39)_b_	12.40(5.15)_b_	113.23(2438)***	0.34	1.54	1.56	0.02
State anger	0.70(1.72)_a_	2.56(4.45)_b_	3.17(5.25)_b_	21.79(2444)***	0.09	0.63	0.84	0.21
Trait anger	4.35(3.11)_a_	10.81(5.35)_b_	9.68(6.42)_b_	115.99(2438)***	0.35	1.60	1.32	0.28
Impulsivity	34.45(10.57)_a_	45.50(15.13)_b_	51.03(12.86)_c_	80.32(2387)***	0.29	1.43	0.94	0.48
Personality limit	3.02(2.15)_a_	6.00(3.07)_c_	7.84(2.83)_b_	170.31(2432)***	0.44	2.00	1.23	0.76
Anti‐social personality	1.12(1.69)_a_	3.35(3.11)_b_	3.04(2.24)_b_	47.14(2438)***	0.18	1.02	0.87	0.14

*Note:* Means with different subscripts differ at the *p* = 0.05 level by Tukey's post hoc test; ***p < 0.001; LRR = low risk of recidivism; MRR = moderate risk of recidivism; HRR = high risk of recidivism; mild psychological IPV, severe psychological IPV, mild physical IPV and severe physical IPV, measured on the CTS2 scale; general physical aggression and general verbal aggression, measured by the AQ aggression questionnaire; alcohol consumption, measured by the AUDIT questionnaire; primary and secondary psychopathy, measured by the LSRP; state anger and trait anger, measured by the STAXI2; impulsivity, measured by the Plutchik impulsivity scale; borderline personality, measured by the SCIDII.

Post hoc analyses revealed statistically significant differences in the scores for all indicators between profiles, especially between the LRR in relation to the MRR and HRR groups. In addition, in five indicators: (a) Mild Psychological IPV (LRR: M = 9.21 SD = 12.20 vs. MRR: M = 26.74, SD = 23.32 vs. HRR: M = 53.24, SD = 30.16); (b) Severe Psychological IPV (LRR: M = 0.70, SD = 1.69 vs. MRR: M = 2.91, SD = 3.77 vs. HRR: M = 30.75, SD = 9.75); (c) Mild Physical IPV (LRR: M = 1.55, SD = 3.24 vs. MRR: M = 4.57, SD = 7.36 vs. HRR: M = 9.08, SD = 10.88); (d) Impulsivity (LRR: M = 34.45, SD = 10.57 vs. MRR: M = 44.50, SD = 15.13 vs. HRR: M = 51.03, SD = 12.86); and (e) borderline personality (LRR: M = 3.02, SD = 2.15 vs. MRR: M = 6.00, SD = 3.07 vs. HRR: M = 7.84, SD = 2.83). Significant differences were also found between the MRR and HRR cases.

### 2.3 Survival Analyses on 15‐Year Recidivism Based on Risk Profile

First, the change between pre‐ and post‐treatment in IPV levels was analysed based on membership in the latent profiles. Statistically significant reductions were observed for mild psychological IPV (*Omnibus F* = 49.5, *p <* 0.001), severe psychological IPV (*Omnibus F* = 286, *p <* 0.001) and mild physical IPV (*Omnibus F* = 19.9, *p <* 0.001). The largest differences from pre‐ to post‐treatment were found in the high‐risk recidivism (HRR) group, followed by the moderate‐risk recidivism (MRR) and low‐risk recidivism (LRR) groups. The most substantial change was detected in severe psychological aggression, where the effect size was highest (*η*_*p*_^2^ = 0.61). The treatment programme demonstrated greater efficacy in reducing IPV levels among participants in the MRR and HRR profiles, with the most pronounced reductions observed in the HRR group when compared with the LRR group. The largest effect sizes were obtained for mild psychological IPV (Cohen's *d* = 2.25), severe psychological IPV (*d* = 5.83) and mild physical IPV (*d* = 1.39). Nevertheless, it is important to underscore that, although participants in the HRR group showed more significant reductions and larger effect sizes, they continued to exhibit higher posttreatment scores in mild psychological aggression (*F*(2, 359) = 15.78, *p <* 0.001, *η*_*p*_^2^ = 0.08) compared with the LRR group and in severe psychological aggression (*F*(2, 371) = 10.49, *p <* 0.001, *η*_*p*_^2^ = 0.05) compared with both the LRR and MRR groups.

**FIGURE 1:**
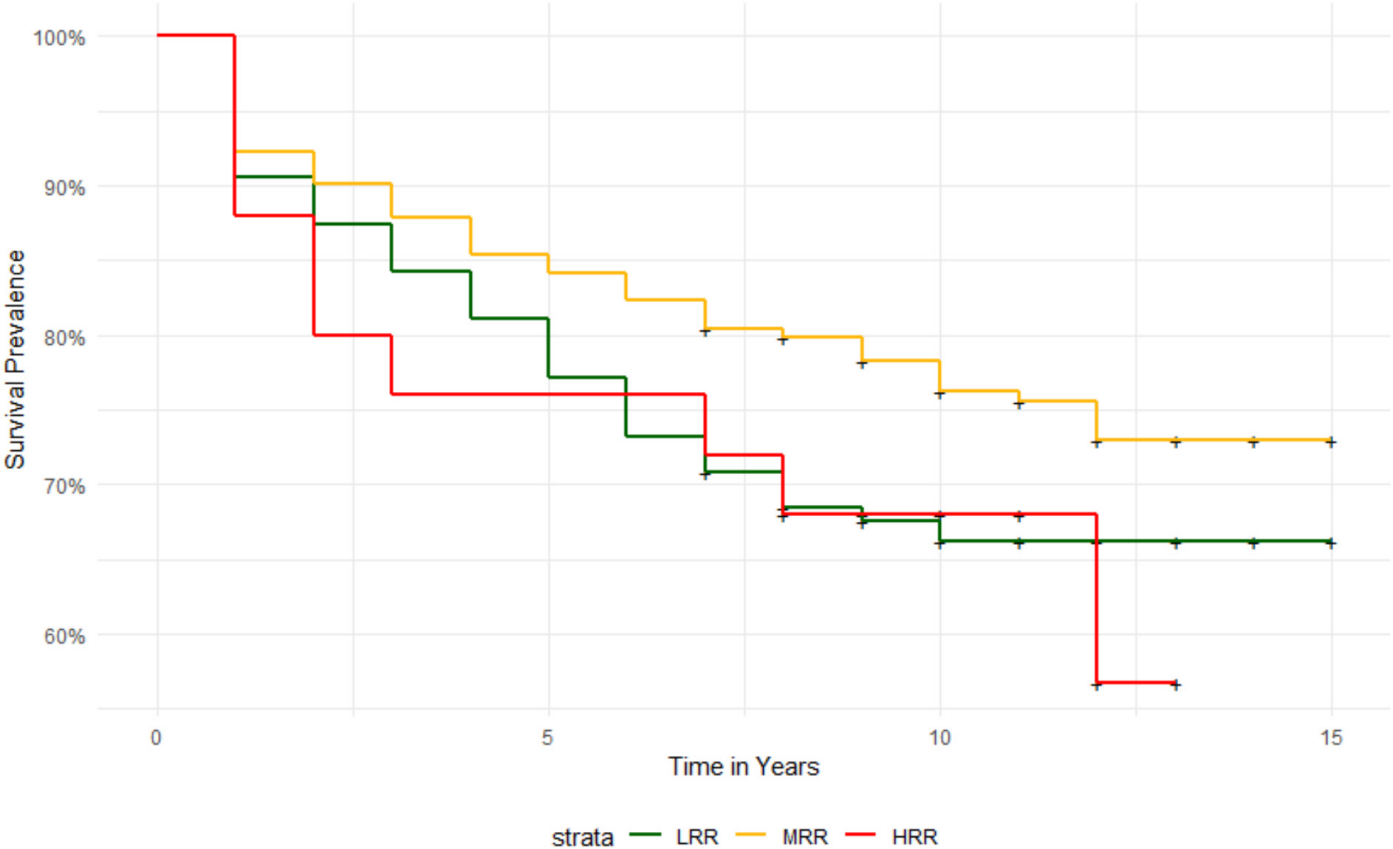
Fifteen‐year survival analysis of recidivism, depending on risk profile (*n* = 478).

A survival analysis was then carried out with the aim of analysing the long‐term recidivism rate for the sample in this study, with a follow‐up of up to 15 years from the initial assessment. Survival analyses indicated that the percentage of recidivism for men in this sample after 15 years of follow‐up was 27% (*n* = 129). In the first 3 years, recidivism totalled 14%. By 5 years of follow‐up, 18% of the men had reoffended. From year 7 of follow‐up, recidivism occurred in 1% of cases per year, and there was no record of recidivism for those who had been followed for more than 12 years (Table 3). When analysing the prevalence of recidivism according to the risk profile (Table 4), it was identified that over the years the profile of IPV perpetrators who had reoffended most frequently was HRR, followed by MRR and LRR. At the end of follow‐up, 23.52% (*n* = 76) of men in the LRR group were identified as having recidivated, versus 32.30% (*n* = 42) of MRRs and 36% (*n* = 9) of HRRs (Figure 1). In addition, it was identified that 33% of cases of recidivism in HRRs (three out of nine) occurred during the first year of follow‐up, whereas the highest frequencies of LRR recidivism were recorded between years 1 and 3 (39 of 76; 51% of the total in the group) and continued to increase gradually until year 12 of follow‐up. Finally, in the medium‐risk group, recidivism maintained high frequencies between years 1 and 6 (34 out of 42; 80% of the total in the group), and the number of cases of recidivism continued to increase between years 1 and 3 until year 10 of follow‐up, at which time no further recidivism was recorded.

**TABLE 3:** Fifteen‐year survival analyses of recidivism after finishing IPV perpetrator treatment (*n* = 478).

Years	No. of risk	No. of recidivism	Survival	SE	95% CI
1	474	40	0.91	0.01	0.89: 0.95
2	434	13	0.88	0.01	0.86: 0.91
3	421	12	0.86	0.02	0.81: 0.87
4	409	12	0.84	0.02	0.79: 0.85
5	397	9	0.82	0.02	0.76: 0.83
6	388	11	0.80	02	0.74: 0.81
7	377	10	0.77	0.02	0.72: 0.80
8	364	6	0.76	0.02	0.71: 0.79
9	296	5	0.74	0.02	0.69: 0.77
10	221	5	0.73	0.02	0.69: 0.77
11	152	1	0.72	0.02	0.69: 0.75
12	87	3	0.70	0.03	0.65: 0.75
13	39	0	0.70	0.03	0.65: 0.75
14	20	0	0.70	0.03	0.65: 0.75
15	4	0	0.70	0.03	0.65: 0.75

**TABLE 4:** Fifteen‐year survival analyses of cases of recidivism depending on the initial risk profiles (*n* = 478).

Years	Low risk (*n* = 323)			Medium risk (*n* = 130)			High risk (*n* = 25)		
	No. of risk	No. of R	Survival	No. of risk	No. of R	Survival	No. of risk	No. of R	Survival
1	322	25	0.92	127	12	0.91	25	3	0.88
2	297	7	0.90	115	4	0.87	22	2	0.80
3	290	7	0.88	111	4	0.84	20	1	0.76
4	283	8	0.85	107	4	0.81	19	0	0.76
5	275	4	0.84	103	5	0.77	19	0	0.76
6	271	6	0.82	98	5	0.73	19	0	0.76
7	265	6	0.80	93	3	0.71	19	1	0.72
8	257	2	0.80	89	3	0.69	18	1	0.68
9	210	4	0.78	70	1	0.68	16	0	0.68
10	156	4	0.76	52	1	0.66	13	0	0.68
11	104	1	0.76	36	0	0.66	12	0	0.68
12	59	2	0.73	22	0	0.66	6	0	0.68
13	26	0	0.73	12	0	0.66	1	1	0.56
14	15	0	0.73	5	0	0.66	1	0	0.56
15	3	0	0.73	1	0	0.66	1	0	0.56

*Note:* No. of R = number of cases of recidivism.

Finally, Cox regression analysis was used to analyse the increased risk of recidivism over the years of follow‐up, according to which risk group the participants belonged to. The results corroborated that MRR (*B* = 1.59, *p* < 0.05) and HRR (*B* = 1.61, *p* < 0.05) had a higher probability of recidivism compared with LRR.

## 3 Discussion

The results of this study confirm the existence of three differentiated subtypes of partner violent men (HRR, MRR and HRR), depending on the frequency and severity of the IPV and the psychopathological deviation identified in the aggressors: levels of general aggression, alcohol consumption, psychopathy, anger, impulsivity, antisocial and borderline personality. The typology proposed by Graña et al. (2014) was therefore replicated. The results indicated that these three subtypes presented statistically significant differences in all indicators included for establishing latent profiles, especially between the LRR subtype (67.6%) and the MRR (27.2%) and HRR (5.2%) subtypes. In addition, significant differences were found between the MRR and HRR subtypes in the three most frequent IPV indicators in this sample: mild psychological aggression, severe psychological aggression and minor physical aggression. Therefore, despite the fact that the MRR and HRR subtypes have similarities in terms of other psychological characteristics analysed, they differ especially in relation to the levels of IPV perpetrated towards their partners. The presence and clinical characteristics of the three subtypes found in this study were consistent with the scientific literature on subtypes of IPV perpetrators (Alexander and Johnson 2023; Cavanaugh and Gelles 2005; Holtzworth‐Munroe et al. 2000; Holtzworth‐Munroe and Stuart 1994; Peters et al. 2023). Empirical evidence of this type of typology has been the predominant alternative in recent years, in an attempt to mitigate the problems of adherence to treatment in these offenders, as well as to improve the effectiveness of the treatment programmes that have been applied to this type of population (Cantos et al. 2019; Redondo, Muñoz‐Rivas, et al. 2023).

Regarding the programme outcomes, the treatment programme showed a greater impact in reducing IPV levels among individuals in the MRR and HRR groups, with significantly greater reductions observed in the HRR group compared with the LRR group. However, it is important to highlight that, despite the HRR group showing more substantial reductions and larger effect sizes, they continued to exhibit higher post‐treatment scores in mild psychological aggression compared with the LRR group and in severe psychological aggression compared with both the LRR and MRR groups. This positions the HRR group at a higher risk for recidivism. Despite their significant improvement and engagement in the program, they remain more aggressive at the conclusion of treatment, consistent with findings from previous studies (Huss and Ralston 2008; Oğuztüzün et al. 2023; Redondo et al. 2019). As to recidivism, there are few studies that have undertaken such prolonged follow‐up and differential analysis of recidivism on the basis of the initial profile of the aggressors. In the present study, survival analyses indicated that the percentage of recidivism after 15 years of follow‐up was 27%, similar to that of other studies (Arias et al. 2013; Loinaz 2014; Verdugo et al. 2025) and that the highest frequency of recidivism occurs in the first year after the participants have been through the psychological treatment programme. This finding is also in line with the findings of other studies (Lila et al. 2019; Verdugo et al. 2025). Total recidivism for HRR was 36%, compared with 32.30% for MRRs and 23.52% for LRRs. These results suggested the need to prolong the treatment programme with aggressors at higher risk. 36% of the HRRs recidivated more frequently in the first year after finishing therapy compared with the other two groups who recidivated less at that point and more in the medium term. Even before starting the treatment process, the HRR group presented higher levels of violence and psychopathological deviation which, despite their participation in therapy, placed them at risk for recidivism. Taking into account these results, it was necessary to increase therapeutic efforts in the groups of most serious aggressors, especially because they were nonimprisoned partner‐violent men. It would be advisable for interventions to be carried out with the most at‐risk aggressors in extremely controlled environments, where emphasis would be placed on altering the eventuality of their violent behaviour and the costs of aggression, in addition to all the content already being worked on with all of them. In the treatment programme followed by the aggressors in this study (Graña et al. 2008), the main intervention modules were based on (1) accepting responsibility for IPV, (2) developing strategies for anger management and fostering empathy with victims, (3) identifying and modifying cognitive distortions that may underlie IPV and (4) providing alternative problem‐solving skills to the use of violence and promoting the development of an assertive and nonaggressive communication style. Probably with lower‐risk aggressors, work on the control of emotions such as anger, training in problem‐solving and assertive forms of communication, together with cognitive work on gender roles and other justifications for the use of violence, could lead to an intervention that would be best adjusted to the profile of lower risk aggressors. However, with the most at‐risk aggressors, it would be necessary to delve even deeper into some of these aspects already included in the treatment programme, as well as incorporating new modules and variables such as borderline personality, antisocial or substance use, variables that have been shown to be significant when classifying the participants in this study that had not yet been included in the treatment programme in a structured and exhaustive manner. In addition, it would be necessary to increase and reinforce as far as possible the monitoring periods in the case of these high‐risk aggressors, in terms of both therapeutic and police/judicial follow‐up.

This study has some limitations that should be considered. The sample was composed only of IPV court‐referred offenders, who had not gone to prison, based on the seriousness of the crimes committed. This should entail a certain degree of caution when generalising the results found. On the other hand, it should be considered that as this is a sample that took part in court‐ordered therapy, its responses may be mediated by social desirability. Participants were referred to therapy by court order and may have thought that their responses would result in legal consequences. However, in previous studies with IPV perpetrators in both open and conventional prison, it was found that, despite the possible existence of social desirability, there was concordance, for example, between the self‐reported violence by IPV perpetrators through CTS2 and the proven facts for which they were convicted (Horcajo‐Gil et al. 2019). Therefore, we can conclude that the variability in the responses of the participants in the different assessment instruments cannot be explained solely by the presence of high levels of social desirability. Finally, it would have been interesting to consider recidivism according to information provided by the victims themselves, in order to improve the reliability of the results found. However, the Spanish judicial system does not allow this, and it was impossible to access this information.

## Conflicts of Interest

The authors declare no conflicts of interest.

## Data Availability

Research data are not shared.
